# Regulation mechanism of curcumin mediated inflammatory pathway and its clinical application: a review

**DOI:** 10.3389/fphar.2025.1642248

**Published:** 2025-08-20

**Authors:** Meihua Liu, Jingyi Wang, Zhen Song, Yong Pei

**Affiliations:** ^1^ Research Center of Emotional Diseases, Shenyang Anning Hospital, Shenyang, China; ^2^ Second Clinical College, China Medical University, Shenyang, China; ^3^ Department of Ophthalmology, The Fourth People’s Hospital of Shenyang Affiliated to China Medical University, Shenyang, China

**Keywords:** curcumin, inflammatory pathways, molecular mechanisms, clinical applications, nano-delivery system

## Abstract

Curcumin, a natural polyphenolic compound derived from *Curcuma longa*, has shown great potential in the prevention and treatment of chronic inflammatory diseases due to its significant antioxidant and anti-inflammatory properties. This article aims to systematically review the anti-inflammatory molecular mechanism, clinical application prospects and challenges of curcumin. By searching the databases of Web of Science, PubMed, Google Scholar and CNKI, and integrating the latest research progress, it was found that curcumin exerted its core anti-inflammatory effects mainly by inhibiting the activation of nuclear factor-κB (NF-κB) signaling pathway, regulating the mitogen-activated protein kinase extracellular signal-regulated kinase (ERK) phosphorylation cascade, and regulating the Janus kinase/signal transducer and activator of transcription (JAK/STAT) pathway. Pharmacological studies have confirmed the therapeutic value of curcumin in a variety of inflammation-related diseases, including neurodegenerative diseases, inflammatory bowel disease, atherosclerosis, diabetes and tumors. Although curcumin has good safety and extensive sources, its inherent low bioavailability severely limits its clinical application. This review points out that combining cutting-edge technologies such as new nano-delivery systems, optimizing the delivery efficiency of curcumin and exploring its anti-inflammatory mechanism in depth are the focus of future research, which is expected to promote it to become a more effective clinical anti-inflammatory drug.

## 1 Introduction

Inflammation is a complex defense response of the body to a variety of pathological stimuli, which involves different types of cells and signaling pathways in the immune system. Although self-limiting inflammation promotes the healing of damaged tissues, an imbalance between the recruitment of inflammatory cells in the body and the clearance of immune infiltrates leads to persistent inflammation, which exceeds the body’s ability to repair itself and triggers serious diseases (Buckley et al., 2015). Chronic inflammation as a central pathological mechanism in cardiovascular disease, cancer, diabetes, chronic kidney disease, steatosis disease, and autoimmune and neurodegeneration diseases, has become a major driver of the global burden of disease (Furman et al., 2019). Epidemiological studies have shown that inflammatory-related diseases account for more than 60% of global mortality, and the incidence is increasing yearly. In recent years, the diagnosis and treatment methods have been improved, but the long-term use of existing anti-inflammatory drugs (such as non-steroidal anti-inflammatory drug and steroids) is often accompanied by serious side effects such as gastrointestinal injury and cardiovascular risk, its clinical application is limited. Therefore, the development of safe and effective therapeutic drugs is imminent. In recent years, natural anti-inflammatory compounds have attracted extensive attention due to their multi-target and low-toxicity characteristics. Among them, plant-derived active ingredients such as curcumin have shown great potential in regulating inflammatory pathways, it provides a new direction for the prevention and treatment of inflammatory diseases (de Lima-Vasconcellos et al., 2026; Gharat et al., 2025; Jiang et al., 2025).

**FIGURE 1:**
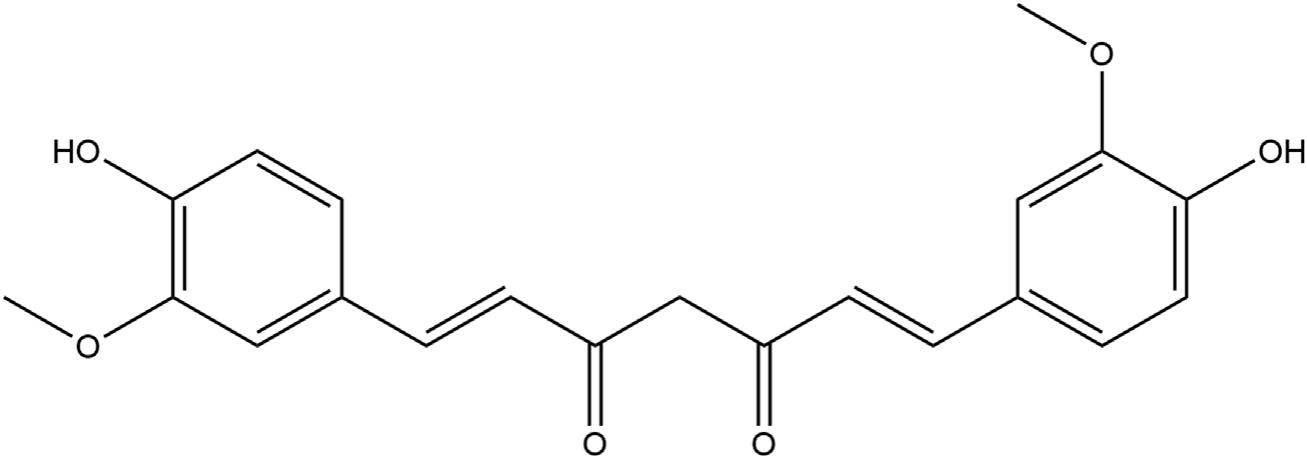
Chemical structure of curcumin.

Curcumin is a natural polyphenolic compound extracted from the rhizome of *Curcuma longa* L., which is widely used in the food industry as a food additive. Its chemical structure is 1,7-bis (4-hydroxy-3-methoxyphenyl) −1,6-heptadiene-3,5-dione (Figure 1), which has anti-inflammatory, anti-oxidation, anti-virus, anti-infection, anti-tumor and other biological activities (He et al., 2015; Abdollahi et al., 2018). Among them, anti-inflammatory properties are considered to be the basis of its multiple biological activities and play an important role in the treatment of diseases (Lestari and Indrayanto, 2014). Studies have shown that curcumin can intervene in the occurrence and development of a variety of chronic inflammatory diseases by regulating key inflammatory signaling pathways such as NF-κB, MAPK, and JAK-STAT, such as rheumatoid arthritis, inflammatory bowel disease and neurodegeneration (Naksuriya et al., 2014). This paper aims to explore the anti-inflammatory mechanism of curcumin and its potential in clinical application, and provide a theoretical basis for the research and application of curcumin.

## 2 Anti-inflammatory molecular mechanism of curcumin

The significant anti-inflammatory activity of curcumin stems from its regulation of multiple key signaling pathways, including NF-κB, MAPK, JAK-STAT, NLRP3 inflammasome and Nrf2/ARE pathways, anti-inflammatory activity (Hanai and Sugimoto, 2009).

### 2.1 Inhibition of NF-κB signaling pathway activation

**FIGURE 2:**
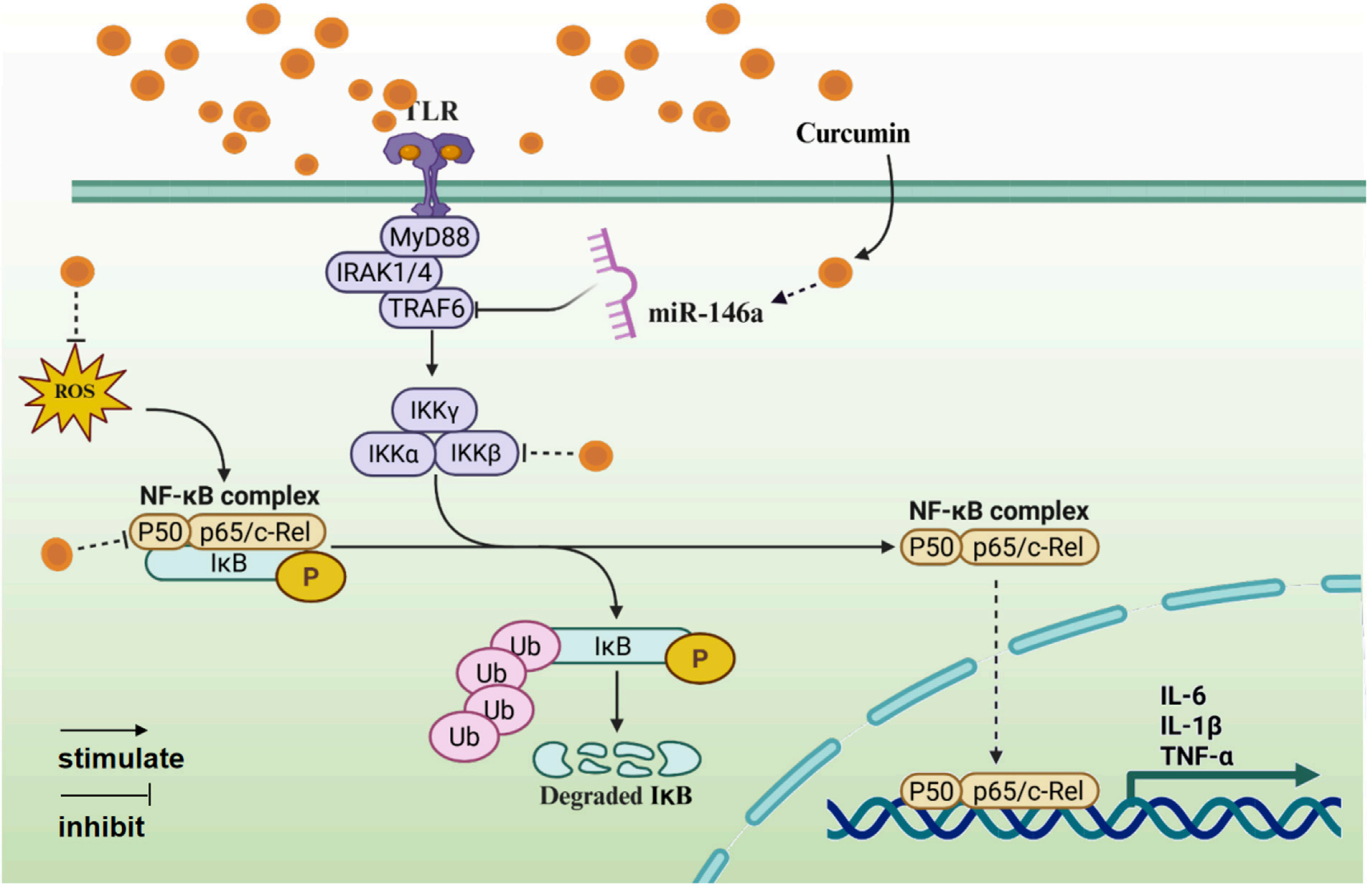
The anti-inflammatory mechanism of curcumin mediated by NF-κB signaling pathway (Created with BioRender.com).

NF-κB is the core regulatory factor of inflammatory response, and its abnormal activation is closely related to chronic inflammatory diseases (Yu et al., 2020). Curcumin interferes with the NF-κB pathway through its multi-target characteristics (Figure 2).

1. Inhibition of IκB kinase (IKK) activity. In the classical pathway, the IKK complex can promote IκBα phosphorylation for its ubiquitinated degradation (Lawrence, 2009; Chen and Greene, 2004). Curcumin inhibits IKKβ subunit activity and blocks IκBα phosphorylation and NF-κb nuclear translocation, thereby inhibiting κBα degradation and NF-κB activation (Zhang et al., 2017).
2. Decreasing transcription factor activity. p65/p50 is a core member of the NF-κB transcription factor family. Curcumin attenuates NF-κB transcriptional activity by blocking its nuclear translocation and inhibiting p65 phosphorylation (Chen et al., 2018).
3. Regulating microRNA. MiR-146a mainly regulates the target genes of NF-κB related pathways, thereby affecting the process of inflammatory response (Zheng et al., 2020). Curcumin can inhibit the NF-κB signaling pathway by upregulating miR-146a expression and targeting TRAF6 (Chen et al., 2021).
4. Antioxidant synergistic effect. Reactive oxygen species (ROS) is an important regulator of NF-κB. Curcumin can inhibit inflammation by scavenging ROS and inhibiting the activation of NF-κB (Das and Vinayak, 2012; Saleem et al., 2025).

### 2.2 Blocking the cascade reaction of MAPK signaling pathway

**FIGURE 3:**
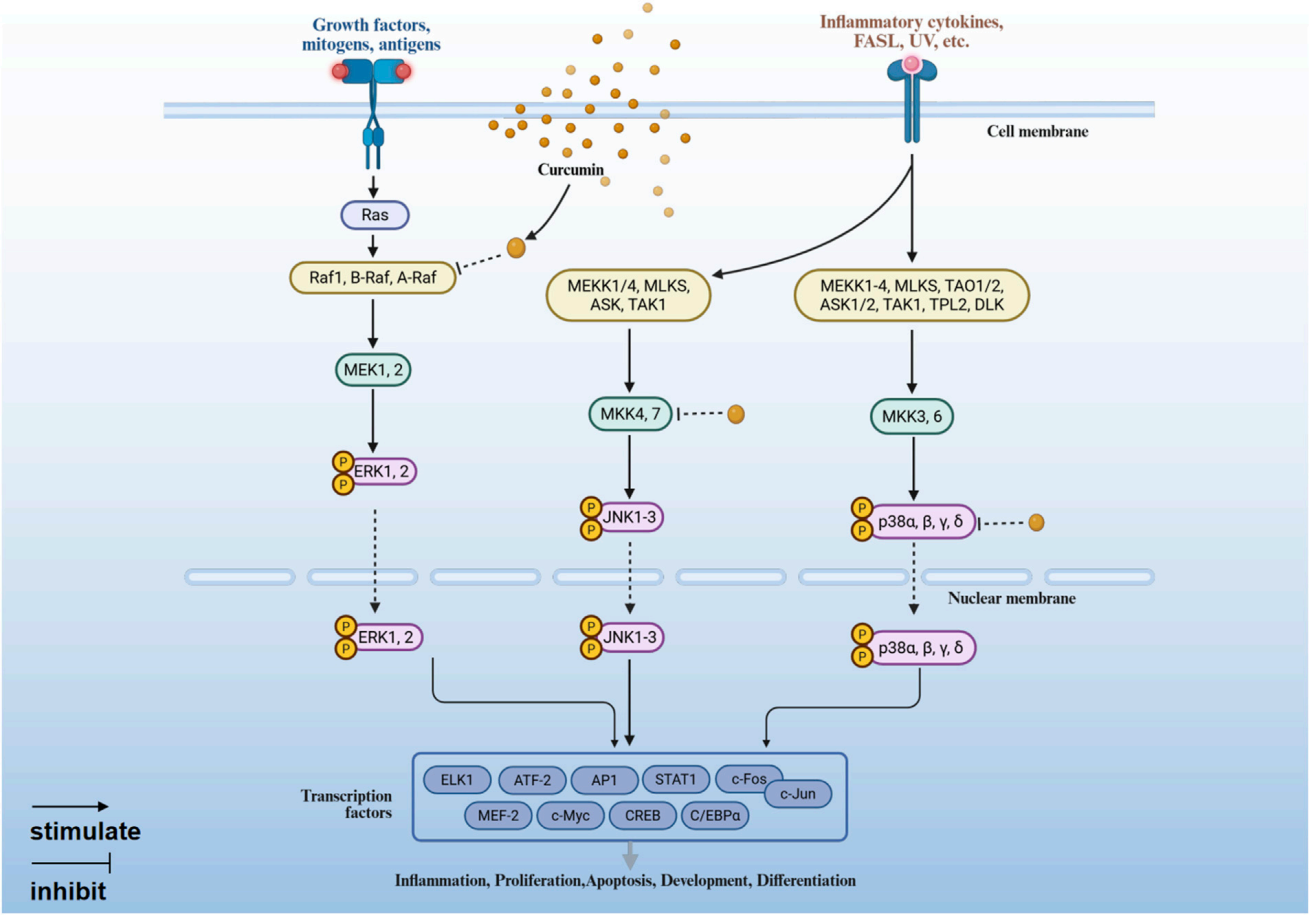
The anti-inflammatory mechanism of curcumin mediated by MAPK signaling pathway (Created with BioRender.com).

MAPK pathway regulates the expression of pro-inflammatory genes through ERK, JNK and p38 three branches (Zeyen et al., 2022; Hepworth and Hinton, 2021; García-Hernández et al., 2021). Curcumin can inhibit the activation of ERK, JNK and p38 MAPK, thereby inhibiting cell proliferation, inducing apoptosis and reducing inflammation (Figure 3).

1. Inhibition of the ERK signaling pathway. Raf-1 is involved in the activation of ERK1/2. Curcumin inhibits the phosphorylation of Raf kinase, thereby blocking MEK1/2 from phosphorylating ERK1/2, which in turn inhibits the activation of ERK1/2 (Sun et al., 2017; Wu, 2012).
2. Inhibition of JNK signaling pathway. Curcumin is able to target MKK4/7 kinases, inhibit JNK phosphorylation, and reduce c-Jun mediated inflammatory gene transcription (Yu et al., 2018).
3. Inhibition of p38 MAPK signaling pathway. Curcumin exerts anti-inflammatory effects by inhibiting the activation of P38 MAPK through upregulation of the MAPK phosphatase MKP-1 (Konduru et al., 2017).

### 2.3 Inhibition of JAK-STAT signaling pathway

**FIGURE 4:**
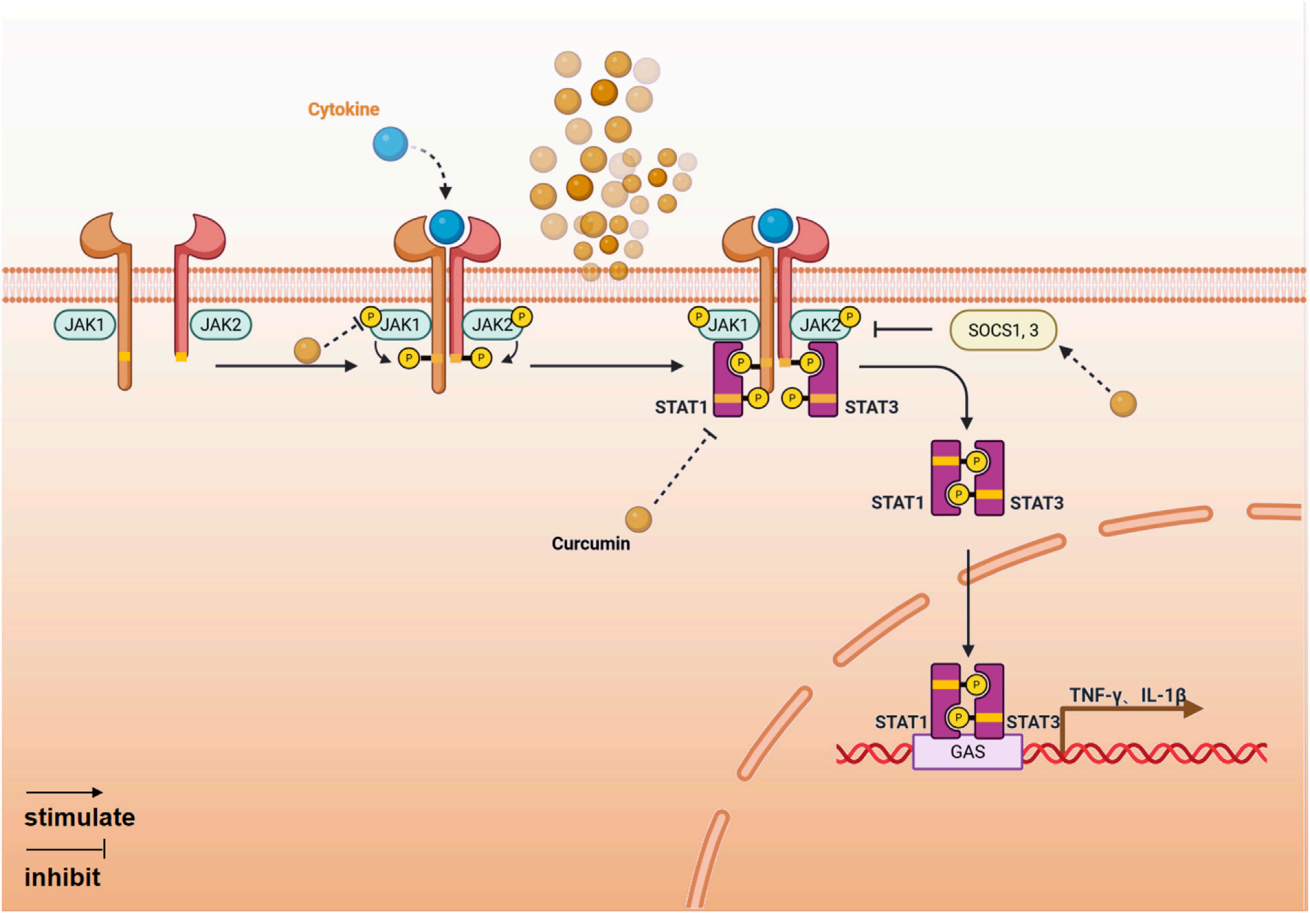
The anti-inflammatory mechanism of curcumin mediated by JAK-STAT signaling pathway (Created with BioRender.com).

The JAK-STAT signaling pathway is one of the most crucial pathways for regulating immune cell inflammation. It responds to various pro-inflammatory cytokines by transducing signals from type I and type II cytokine receptors (O'Shea et al., 2013). Curcumin can inhibit JAK-STAT signal transduction by directly targeting JAK kinase activity and modulating STAT function (Figure 4).

1. Inhibition of JAK and STAT phosphorylation. Studies have shown that curcumin reduces the level of JAK1/2 and STAT1/3 phosphorylation, thereby inhibiting the expression of pro-inflammatory factors mediated by the JAK-STATs signaling pathway (Liu et al., 2025; Zhang et al., 2024).
2. Enhance the expression of negative feedback regulatory proteins. The SOCS family is a negative regulatory protein that inhibits the activity of the JAK-STAT signaling pathway and regulates the duration of the signal. Curcumin induces the expression of proteins such as SOCS1 and SOCS3, downregulates JAK2-STAT3/STAT6, inhibits the activity of the JAK-STAT signaling pathway, reduces the levels of pro-inflammatory cytokines, increases the levels of anti-inflammatory cytokines, and alleviates inflammation (Zhao et al., 2016).

### 2.4 Regulates NLRP3 inflammasome

**FIGURE 5:**
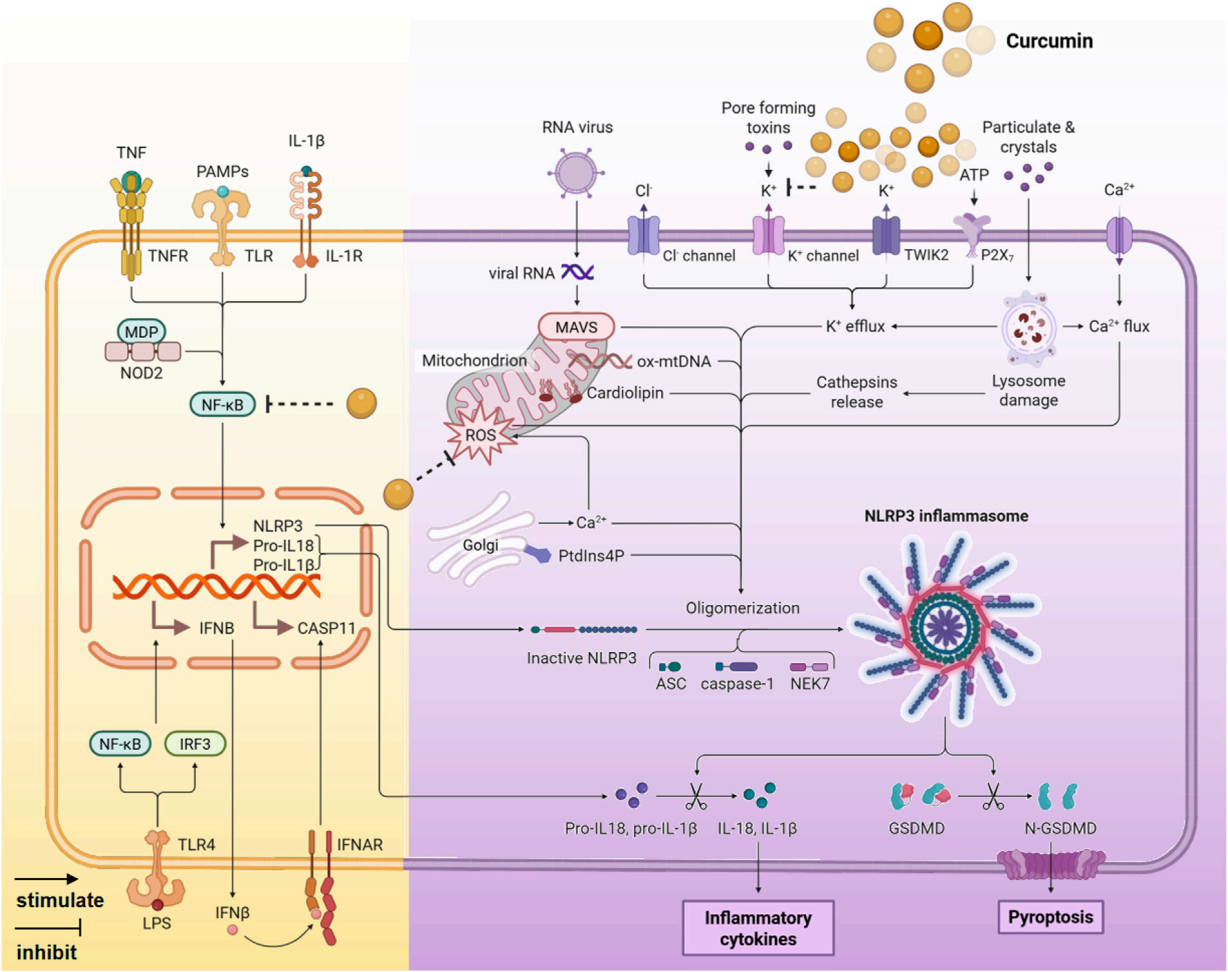
The anti-inflammatory mechanism of curcumin mediated by NLRP3 signaling pathway (Created with BioRender.com).

Excessive activation of the NOD-like receptor protein 3 (NLRP3) inflammasome can lead to pyroptosis and tissue damage (Wang and Hauenstein, 2020). Curcumin specifically inhibits the initiation and assembly of the NLRP3 inflammasome, thereby suppressing the inflammatory cascade (Figure 5).

1. Inhibition of NLRP3 inflammasome activation. The activation of the NLRP3 inflammasome is inhibited by curcumin, which suppresses K^+^ efflux, the downstream spatial localization of mitochondria, ASC dimerization, and speck formation (Gong et al., 2018; Yin et al., 2018).
2. Inhibition of the NF-κB signaling pathway. The NF-κB signaling pathway is a crucial upstream regulator of NLRP3 inflammasome activation. Curcumin inhibits NF-κB activation by preventing the degradation of IκBα and reducing the phosphorylation levels of NF-κB subunits (p65 and p50), thereby hindering the activation of the NLRP3 inflammasome (Li et al., 2025; Chen et al., 2019; Lan et al., 2023).
3. Antioxidant effect. Curcumin can increase SOD activity, reduce oxidative stress, and eliminate the oxidative damage incentives of NLRP3 activation (Yang et al., 2023).

### 2.5 Activation of Nrf2/ARE antioxidant pathway

The Nrf2/ARE pathway, a classical oxidative stress signaling pathway, is closely associated with oxidative stress and inflammation in the body. Curcumin facilitates the nuclear translocation of Nrf2, upregulates the expression of antioxidant genes such as HO-1 and NQO-1, and reduces LPS-induced oxidative damage as well as the secretion of IL-1β and TNF-α. Gene knockout experiments have confirmed that the absence of Nrf2 significantly weakens the anti-inflammatory effects of curcumin, indicating that this pathway is a key target for its action (Zhao et al., 2011; Jiang et al., 2020).

Curcumin exerts regulatory effects during the initiation, development, and chronic stages of inflammation through its multi-target synergistic actions. It inhibits pro-inflammatory pathways such as NF-κB, MAPK, and NLRP3, while activating antioxidant pathways like Nrf2. Additionally, it modulates cytokine networks and immune cell functions (Vallée et al., 2019; Yin et al., 2018; Zhang X. Y. et al., 2019). This multifaceted mechanism gives it potential in treating various diseases, including arthritis, intestinal inflammation, and neuroinflammation.

## 3 Pharmacologic studies of curcumin in inflammatory diseases

**FIGURE 6:**
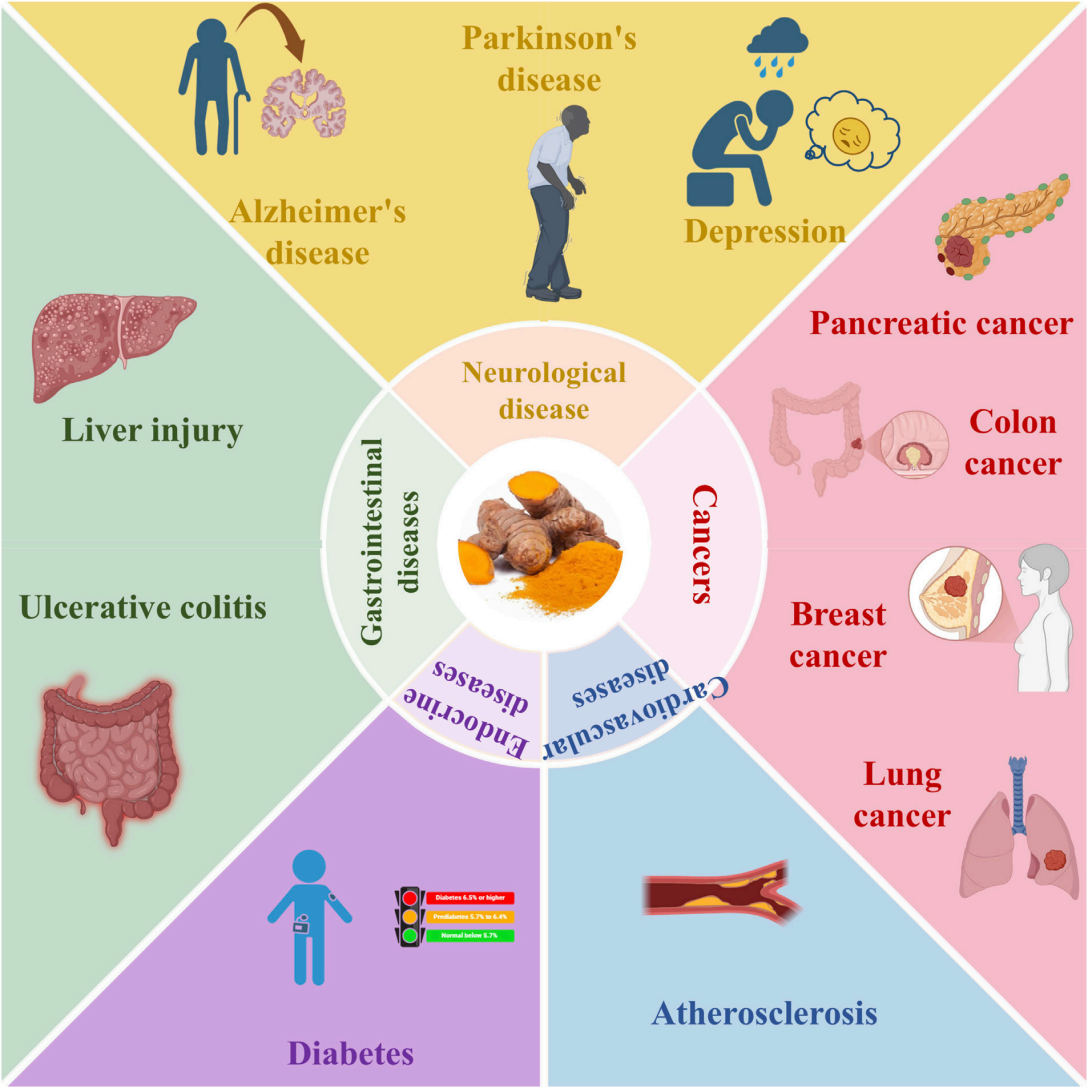
Clinical effect of curcumin (Created with BioRender.com).

*Curcuma longa* L. as a traditional Chinese medicine with the same source of food and medicine, its medicinal use was first recorded in the Tang Dynasty “Newly Revised Materia Medica”, which recorded that it is “the main cardiac and abdominal stagnation, propulsion and resistance, under the gas to break the blood” and other efficacy. Modern research has further confirmed that turmeric has anti-hepatic injury, regulating blood pressure and blood lipids, menstruation and pain relief and other pharmacological effects. Curcumin extracted from turmeric, as a natural dual-use ingredient, has been proven to have anti-inflammatory (Zeng et al., 2022), anti-oxidative stress (Ghareghomi et al., 2021), anti-tumor proliferation (Zhao et al., 2023), mucosal protection (Sheethal et al., 2020), broad-spectrum antimicrobial (Reddy et al., 2020), regulation of lipid metabolism (Qin et al., 2017), improvement of insulin resistance (Heshmati et al., 2021), promotion of bile excretion (Hong et al., 2022), and hepatopoietic cell protection (Bavarsad et al., 2019) and other diversified bioactivities. It has become a hot spot of natural medicine research at home and abroad. Based on the above wide range of pharmacological effects, curcumin has shown potential application value in the treatment of various inflammation-related diseases (Figure 6).

### 3.1 The role of curcumin in neurodegenerative diseases

Neurodegenerative diseases, including Alzheimer’s disease (AD), Parkinson’s disease (PD), and epilepsy, are characterized by the progressive necrosis or functional loss of nerve cells. Studies have shown that, besides the reduction of antioxidants and the oxidative damage to proteins, DNA, and lipids (Lee et al., 2012; Höhn and Grune, 2013), chronic inflammatory responses are also significant contributors to disease progression (Süβ et al., 2021) (Table 1).

**TABLE 1:** The role of curcumin in neurodegenerative diseases.

Disease	Dosages	Minimal active concentration	Positive control drug	Model	Mechanism	References
Alzheimer’s Disease	150 and 300 mg/kg	150 mg/kg	--	5 × FAD Transgenic Mice	Inhibition of BACE1 expression and reduction of Aβ deposition	Zheng et al. (2017)
	0, 1.25, 5.0 and 20.0 µM	5.0 µM	--	SH-SY5Y	Inhibition of GSK-3β-mediated PS1 activation reduces Aβ production	Xiong et al. (2011)
	100 mg/kg	--	--	C57BL/6J male mice	Regulating GSK3β/Wnt/β-catenin and CREB/BDNF pathways by targeting PI3K/Akt	Lou et al. (2024)
Parkinson’s Disease	0, 0.1, 0.5 and 1 µM	0.1 µM	--	PC12 cells	Inhibition of oxidative stress and mitochondrial cell death pathways, inhibition of A53T mutant α-synuclein-induced cell death	Liu et al. (2011)
	80 mg/kg	--	Levodopa	Adult male Swiss albino mice	Improves α-synuclein levels and reduces lewis and inflammatory mediator levels	Motawi et al. (2020)
	1, 2 and 4 μM	1 μM	Levodopa	SH-SY5Y cells	--	Guo et al. (2025)
Epilepsy	200 mg/kg	--	--	Male ICR mice	Reduce the levels of GFAP, eNOS and HO-1 in hippocampus, inhibit the expression of reactive astrocytes, and prevent the death of hippocampal cells	Shin et al. (2007)
	0.4, 2, and 10 mg/kg	0.4 mg/kg	--	C57BL/6 mice	Upregulation of NF1X level increased the proliferation activity of neural progenitor cells (NPC)	Lee et al. (2023)

Curcumin exerts neuroprotective effects through dual mechanisms: on one hand, it directly scavenges free radicals or binds with Cu^2+^ and Fe^2+^ to form complexes, thereby alleviating metal ion-induced oxidative damage; on the other hand, it inhibits the activity of NF-κB, lipoxygenase, and cyclooxygenase-2, thus mitigating neuroinflammation (Kant et al., 2014; Lee et al., 2013). In the lipopolysaccharide (LPS) induced BV2 microglial cell model, curcumin reduced neuroinflammation by inhibiting the TLR4/NF-κB pathway, downregulating TREM2 expression, and promoting the conversion of M1-type microglia to the anti-inflammatory M2-type (Zhang J. et al., 2019). However, this study only conducted cell experiments and lacked animal experiments. In Parkinson’s disease models, curcumin reduced the levels of reactive oxygen species (ROS) induced by mutant α-synuclein in PC12 cells, inhibited mitochondrial damage and the activation of caspase-9/3, and improved cytotoxicity (Liu et al., 2011). Additionally, curcumin decreased oxidative stress markers in the hippocampal tissue of pilocarpine-induced epilepsy models and prevented hippocampal neuronal loss (Noor et al., 2012). This study confirms the antiepileptic effects of curcumin through animal experiments, but its limitation lies in being limited to demonstrating therapeutic efficacy without revealing the underlying mechanisms, resulting in compromised depth of the conclusions. These effects suggest that anti-inflammatory, antioxidant, and inhibition of abnormal protein aggregation are the core mechanisms by which curcumin intervenes in neurodegenerative diseases.

### 3.2 The role of curcumin in inflammatory bowel disease

Inflammatory bowel disease (IBD) includes ulcerative colitis (UC) and Crohn’s disease (CD), characterized pathologically by intestinal oxidative stress, nitrosative stress, leukocyte infiltration, elevated levels of pro-inflammatory cytokines (Th1-type cytokines IL-12, IFN-γ, TNF-α, IL-1, and Th17-type cytokine IL-17), and decreased levels of anti-inflammatory cytokines (Th2-type cytokines IL-4, IL-5, IL-10) (Table 2) (Zhang et al., 2006).

**TABLE 2:** The role of curcumin in inflammatory bowel disease.

Disease	Dosages	Minimal active concentration	Positive control drug	Model	Mechanism	References
Inflammatory bowel disease	100 and 200 mg/kg	100 mg/kg	--	BALB/c male mice	Inhibition of SphK1/NF-κB signaling pathway activation	Zhang et al. (2025)
	100 and 300 mg/kg	100 mg/kg	--	Sprague-Dawley rats	Regulating the balance of Treg/Th17 and the secretion of related cytokines	Guo et al. (2022)
	40 mg/kg	--	Mesacol tablet	Rat	--	Desai and Momin (2020)

In a sodium sulfate-induced ulcerative colitis model, oral administration of curcumin (200 mg/kg) effectively inhibited the SphK1/NF-κB signaling pathway. This inhibition alleviated intestinal mucosal damage, promoted the repair of goblet cells, and reduced serum levels of inflammatory factors (TNF-α, IL-1β, IL-8) as well as myeloperoxidase (MPO) in colon tissue. Curcumin demonstrated significant therapeutic effects against ulcerative colitis (Zhang et al., 2025). Additionally, curcumin regulated the Treg/Th17 cell balance, increased the secretion of the anti-inflammatory cytokine IL-10, and decreased the expression of the pro-inflammatory cytokine IL-17A, thereby mitigating intestinal inflammatory responses (Guo et al., 2022).

### 3.3 The role of curcumin in atherosclerosis

Atherosclerosis (AS) is a disease of the peripheral arteries accompanied by chronic inflammation that can lead to vascular system dysfunction and a variety of diseases (Table 3). Accumulation of macrophages within the arterial wall is a prominent feature of atherosclerotic plaques. Influenced by various stimuli in the plaque microenvironment, macrophages polarize into pro-inflammatory M1 and anti-inflammatory M2 macrophages. Studies found that curcumin (30 μM) inhibited the NF-κB pathway, reduced the differentiation of M1 pro-inflammatory macrophages, decreased the activity of the TLR4/MAPK/NF-κB cascade signaling, and promoted the transformation of macrophages from a pro-inflammatory phenotype to an anti-inflammatory phenotype (Momtazi-Borojeni et al., 2019). Furthermore, *in vitro* experiments treating RAW264.7 macrophages (M0 and M1 phenotypes) with curcumin analyzed the molecular basis of its anti-atherosclerotic activity. Curcumin activated PPARγ, thereby promoting macrophage polarization from M0/M1 to M2 phenotypes. This polarization increased anti-inflammatory factor expression, suppressed systemic inflammatory responses, and delayed atherosclerotic (AS) progression. Tested concentrations were 0, 6.25, 12.5, and 25 μmol/L (Chen et al., 2014).

**TABLE 3:** The role of curcumin in atherosclerosis.

Disease	Dosages	Minimal active concentration	Positive control drug	Model	Mechanism	References
Atherosclerosis	0, 7.5, 15, and 30 μM	7.5 μM	--	TJP-1 cells	Reduce TLR4 expression and inhibit MAPK/NF-κB pathway	Momtazi-Borojeni et al. (2019)
	0, 6.25, 12.5, and 25 μM	6.25 μM	--	RAW264.7	Activation of IKBα inhibits M1 inflammatory phenotype, and activation of PPARγ polarizes macrophages to M2 phenotype	Chen et al. (2014)

Curcumin, in addition to its anti-inflammatory effects that help delay the progression of AS, can also slow down AS progression by reducing oxidative stress, providing anticoagulant effects, improving lipid metabolism, enhancing glucose metabolism, regulating the proliferation and migration of smooth muscle cells, lowering blood pressure, reducing pathological neovascularization, and protecting endothelial cells. Further research is needed to fully understand these specific mechanisms (Wang et al., 2022).

### 3.4 The role of curcumin in diabetes

In type 1 diabetes, curcumin, as a heme oxygenase-1 (HO-1) inducer, improved blood glucose levels (27.5% lower) and insulin secretion (66.67% higher) in diabetic rats, as well as regulating blood lipids and attenuating lipid peroxidation damage in the pancreas and liver (Abdel Aziz et al., 2012). In type 2 diabetes, curcumin exerts its efficacy by inhibiting oxidative stress and inflammatory response (Nishiyama et al., 2005). At present, there are limited studies on the role of curcumin in this context, and there are few studies on its potential mechanism. In the type 2 diabetic rat model established by high-fat diet feeding combined with intraperitoneal injection of streptozotocin, curcumin (200 mg/kg) lowered fasting blood glucose, improved pancreatic β-cell function, and reduced apoptosis. Mechanistically, it inhibited the inflammatory cascade response and apoptosis by down-regulating pro-inflammatory factors such as IL-1β and IL-6 and pro-apoptotic proteins (Bax and caspase-3), up-regulating antioxidant enzymes (SOD2 and glutathione peroxidase) and the anti-apoptotic protein Bcl-2, and blocking the RAGE/JNK/NF-κB signaling pathway (Qihui et al., 2020) (Table 4).

**TABLE 4:** The role of curcumin in diabetes mellitus.

Disease	Dosages	Minimal active concentration	Positive control drug	Model	Mechanism	References
Diabetes	10 mg/kg	--	--	Rats	--	Abdel Aziz et al. (2012)
	200 mg/kg	--	--	Sprague-Dawley rats	Inhibition of phosphorylated JNK and NF-κB protein expression, blocking RAGE/JNK/NF-κB signaling pathway	Qihui et al. (2020)
	500 mg/person	--	Zinc	Diabetic patients	--	Karandish et al. (2021)

### 3.5 The role of curcumin in tumor

Inflammatory mediators play an important role in tumors. Recent basic and clinical studies have shown the efficacy of curcumin as a multi-target intracellular signaling pathway modulator in ameliorating tumors such as lung, liver, gastric, colorectal, pancreatic and cervical cancers (Table 5).

**TABLE 5:** The role of curcumin in tumor.

Disease	Dosages	Minimal active concentration	Positive control drug	Model	Mechanism	References
Tumor	0, 5, 10, 20 µM	20 µM	--	Human PSCs	Inhibition of IL-6/ERK/NF-κB axis	Li et al. (2020)
	0, 5, 10, 20 µM	5 µM	--	Human PSCs	Inhibition of ROS/ERK/NF-κB signaling pathway	Cao et al. (2016)
	30 mg/rat	--	--	BALB/c mice	Regulating the NF-KB/UPS axis	Zhang et al. (2022)

Inflammation is closely related to tumor development, and curcumin, as a multi-target signaling modulator, shows intervention potential in a variety of tumors (Coussens and Werb, 2002). Treatment of pancreatic stellate cells with curcumin reduced pancreatic stellate cell activation and migration and blocked tumor-stromal crosstalk and pancreatic cancer metastasis by inhibiting the IL-6/ERK/NF-κB signaling axis (Li et al., 2020). In ovarian cancer, it reduced fascin protein expression and inhibited tumor cell recurrence and metastasis by inhibiting the JAK/STAT3 pathway (Kim et al., 2020). In addition, curcumin is an inhibitor of the transcription factor NF-κB and downstream gene products (Hasima and Aggarwal, 2012). Curcumin ameliorated skeletal muscle atrophy and mitochondrial dysfunction in breast cancer models by modulating the NF-κB/ubiquitin-proteasome system (UPS) axis, suggesting its therapeutic value in tumor-related complications (Zhang et al., 2022). Although a large number of *in vitro*/animal models have revealed the multi-target anti-tumor mechanism of curcumin, it lacks clinical experimental data verification. At present, the clinical research on the anti-tumor effect of curcumin is limited by small samples, short-cycle trials and inconsistent preparations, resulting in a weak clinical transformation.

### 3.6 Other effects

In addition to the above pharmacological effects, curcumin also plays a role in the prevention and treatment of obesity. It enhanced triacylglycerol lipase activity and reduced the level of low-density lipoprotein by activating the peroxisome proliferator-activated receptor γ (PPAR-γ) signaling pathway, thereby regulating lipid metabolism and improving dyslipidemia (Lee et al., 2020). In the field of autoimmune diseases, curcumin showed potential intervention value in rheumatoid arthritis, multiple sclerosis, and systemic lupus erythematosus. Its mechanism of action mainly involved inhibiting the proliferation of T-cells, B-cells, and dendritic cells, and regulating the levels of a variety of cytokines, thereby inhibiting excessive immune responses (Yang et al., 2019). Although curcumin has shown potential in a variety of disease models, current experimental studies generally design hard wounds and lack positive drug controls. This defect seriously hinders its transformation into clinical practice.

## 4 Safety and toxicity

Curcumin is approved by the U.S. Food and Drug Administration (FDA) as a “Generally Recognized as Safe” (GRAS) substance, and its safety has been demonstrated in multiple studies. The compound has demonstrated therapeutic potential for a wide range of diseases in a wide range of doses with very low risk of toxicity. Cellular experiments showed that although high concentrations of curcumin might have some inhibitory effects on the proliferation and viability of normal cells, the threshold for safe concentrations was much higher than conventional therapeutic doses (Hollborn et al., 2013).

Human clinical studies showed that curcumin exhibited good tolerance even when administered orally at a dose of 6 g/day for 4–7 weeks, with only a few cases reporting mild side effects such as itching, reddening of the tongue, tachycardia, or gastrointestinal disturbances (Ryan et al., 2013). Animal experiments further demonstrated that oral administration of curcumin during pregnancy was not significantly toxic to the mother and fetus. However, high doses (approximately 1,000 mg/kg body weight) resulted in a mild decrease in weight gain in F2 pups, suggesting that potential intergenerational effects required further investigation for long-term high-dose use (Ganiger et al., 2007). Overall, the available evidence supports the high safety profile of curcumin in human applications, especially at routine therapeutic doses, with almost negligible toxic side effects.

## 5 Clinical translational challenges and solutions

**FIGURE 7:**
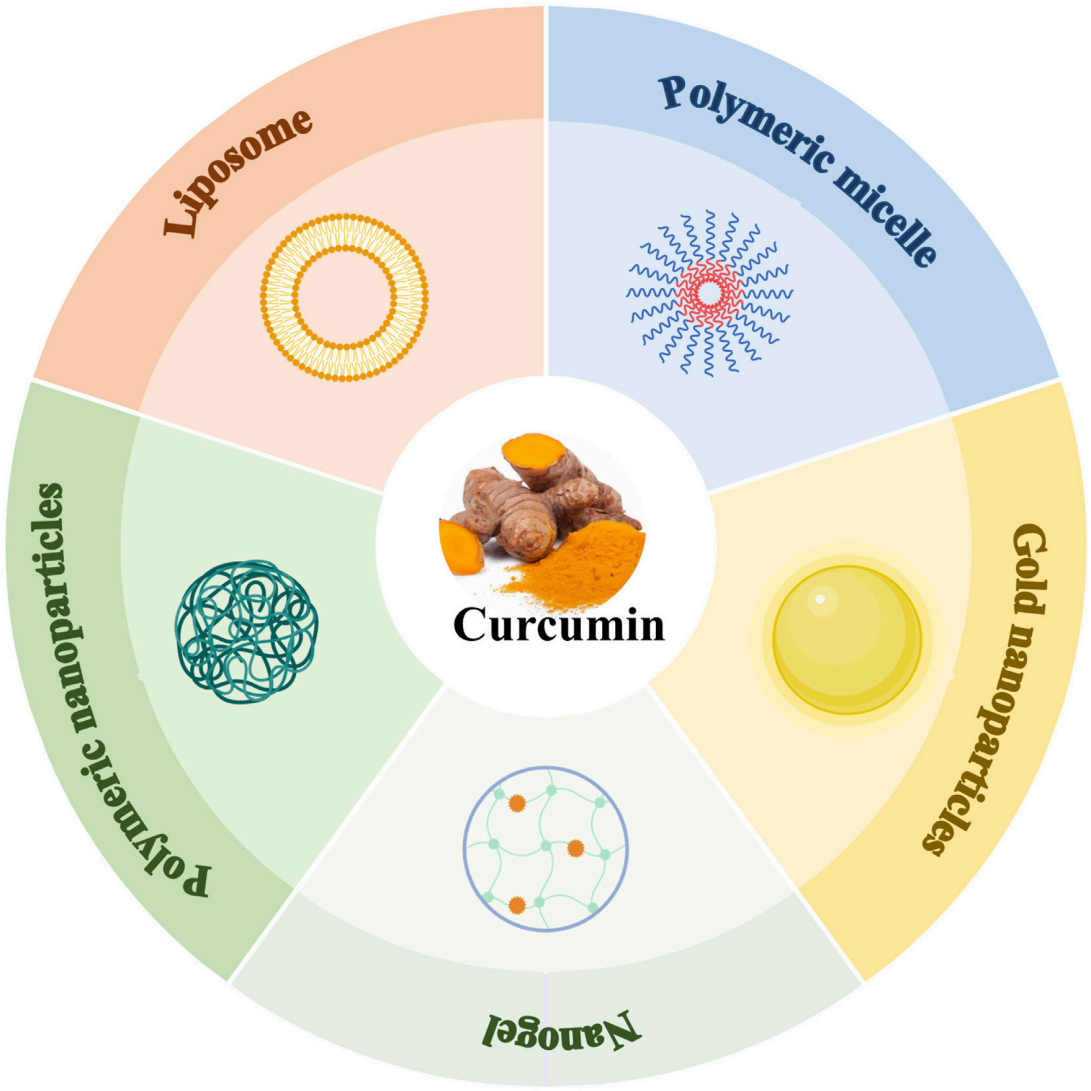
The main nano-preparations of curcumin (Created with BioRender.com).

Although curcumin exhibits a wide range of safety and pharmacological activities, its clinical translation still faces significant challenges, with low bioavailability at the core of the problem. The poor water solubility and inefficient gastrointestinal absorption of curcumin make it difficult to reach effective therapeutic concentrations in the body. To address this problem, the recent development of nanomedicine delivery systems has provided an innovative pathway to break through this limitation by significantly improving its pharmacokinetic properties through carrier modification (Figure 7).

### 5.1 Curcumin liposomes

Since curcumin is a difficult drug to dissolve in water, liposomes were prepared to improve the water solubility and bioavailability of curcumin. The DSPE-PEG2000 modified liposomes constructed by film hydration method had a particle size of about 110–116 nm and an encapsulation rate of 62.5%. Further surface modification by anti-transferrin antibody could specifically recognize the blood-brain barrier transferrin receptor and significantly improve the uptake in the blood-brain barrier cell model, which has potential therapeutic effects in Alzheimer’s disease (Mourtas et al., 2014). Another study utilized rabies virus glycoprotein-derived peptide (RDP)-modified liposomes with a particle size of approximately 100 nm and an encapsulation rate of greater than 85%, which was able to delay the release of Cur compared to the cumulative release degree of ordinary curcumin liposomes (Xiang et al., 2015).

### 5.2 Curcumin solid dispersions

The solid dispersion technology significantly improved curcumin solubilization performance through the solubilizing effect of the carrier material. The solid dispersion with octenyl succinate hydroxypropyl phytoglycogen as the carrier showed a solubility of 1,462.2 μg/mL in phosphate buffer, which was 16,000 times enhancement compared to curcumin, and the 15 min dissolution reached 50% (Xie and Yao, 2020). When Utage^®^ polyacrylic acid resin and hydroxypropyl methyl cellulose were used as the composite carrier, the cumulative drug release could reach more than 90%, which was a 9.7 times enhancement over the free drug and significantly increased the solubility of curcumin (Zhang X. Y. et al., 2019).

### 5.3 Curcumin polymer nanoparticles

Curcumin polymer nanoparticles were prepared by emulsification solvent volatilization method using polylactic acid/hydroxyacetic acid copolymer as a carrier with an average particle size of about 100 nm, encapsulation rate of (81.63 ± 1.96)%, and drug loading capacity of (4.55 ± 0.15)%. *In vivo* studies in rats showed that compared to the free drug, the nanoparticles increased the area under the concentration time curve (AUC) by 2.6 times, prolonged the mean residence time (MRT) by 1.7 times, and the brain drug AUC _(0-t)_ increased from (31.33 ± 6.38) μg·g^-1^·h^-1^ to (45.39 ± 2.08) μg·g^-1^·h^-1^, significantly improving drug distribution in the central nervous system (Ye et al., 2017).

### 5.4 Curcumin polymer micelles

Self-assembled curcumin micelles based on hydroxypropylated debranched starch showed an encapsulation efficiency of 70.3% and a drug loading of 5.33% when the amylose content was at its lowest, with water solubility increased by 300 times compared to the raw drug. *In vitro* release experiments demonstrated a cumulative release of 78% at 6 h, while free drug showed almost no release. Cytotoxicity tests indicated that within the concentration range of 62.5–1,000 μg/mL, the survival rate of Caco-2 cells remained above 90%, and hemolysis was below 5%, exhibiting good biocompatibility and safety (Zhi et al., 2021).

## 6 Conclusion

Curcumin, a natural polyphenolic compound, exhibits broad research and application prospects due to its multi-dimensional regulatory effects on several key inflammatory pathways, including NF-κB, MAPK, JAK-STAT, and the NLRP3 inflammasome, as well as its potential therapeutic value in various inflammation-related diseases such as neurodegenerative disorders, inflammatory bowel disease, atherosclerosis, diabetes, and cancer.

However, its clinical application faces significant bottlenecks, primarily manifested in its inherent extremely low water solubility, poor bioavailability, and unfavorable pharmacokinetic profile. These issues severely limit the realization of its therapeutic potential. To overcome these core barriers, future research urgently needs to focus on several key directions: First, studies on novel delivery systems such as nanoliposomes, solid dispersions, polymeric nanoparticles, and micelles should be deepened, with an emphasis on optimizing the targeting specificity of carrier materials. This aims to significantly enhance curcumin’s water solubility and bioavailability, prolong its circulation time *in vivo*, and precisely increase its drug concentration in target tissues. Second, more rigorously designed, high-quality clinical studies must be conducted to provide conclusive evidence validating curcumin’s actual efficacy and safety in specific diseases. Simultaneously, multi-omics technologies including genomics, transcriptomics, proteomics, and metabolomics should be integrated to deeply analyze curcumin’s mechanism of action within complex disease networks, particularly its synergistic effects with other molecules or pathways. By comprehensively advancing these strategies, it is expected to overcome the current limitations, accelerate the transformation of curcumin from a natural product into a safe and effective clinical drug, and ultimately provide innovative therapeutic solutions for preventing and treating inflammation-related diseases.
